# Biotechnological Interventions in Tomato (*Solanum lycopersicum*) for Drought Stress Tolerance: Achievements and Future Prospects

**DOI:** 10.3390/biotech11040048

**Published:** 2022-10-19

**Authors:** Ram Krishna, Waquar Akhter Ansari, P. S. Soumia, Akhilesh Yadav, Durgesh Kumar Jaiswal, Sudhir Kumar, Achuit Kumar Singh, Major Singh, Jay Prakash Verma

**Affiliations:** 1 Institute of Environment and Sustainable Development, Banaras Hindu University, Varanasi 221005, India; 2 ICAR-Indian Institute of Vegetable Research, Varanasi 221305, India; 3 ICAR Indian Council of Agriculture Research-Directorate of Onion and Garlic Research, Pune 410505, India; 4 Department of Plant Science, University of California, Davis, CA 95616, USA; 5 Department of Botany, Savitribai Phule Pune University, Pune 411007, India; 6 Soil Mircobiology Lab, Department of Soil Science, Pici Campus, Federal University of Ceara, Fortaleza 60020, Brazil

**Keywords:** drought, tomato, *Solanum lycopersicum*, transgenic, antioxidants, antioxidative enzymes

## Abstract

Tomato production is severely affected by abiotic stresses (drought, flood, heat, and salt) and causes approximately 70% loss in yield depending on severity and duration of the stress. Drought is the most destructive abiotic stress and tomato is very sensitive to the drought stress, as cultivated tomato lack novel gene(s) for drought stress tolerance. Only 20% of agricultural land worldwide is irrigated, and only 14.51% of that is well-irrigated, while the rest is rain fed. This scenario makes drought very frequent, which restricts the genetically predetermined yield. Primarily, drought disturbs tomato plant physiology by altering plant–water relation and reactive oxygen species (ROS) generation. Many wild tomato species have drought tolerance gene(s); however, their exploitation is very difficult because of high genetic distance and pre- and post-transcriptional barriers for embryo development. To overcome these issues, biotechnological methods, including transgenic technology and CRISPR-Cas, are used to enhance drought tolerance in tomato. Transgenic technology permitted the exploitation of non-host gene/s. On the other hand, CRISPR-Cas9 technology facilitated the editing of host tomato gene(s) for drought stress tolerance. The present review provides updated information on biotechnological intervention in tomato for drought stress management and sustainable agriculture.

## 1. Introduction

Cultivated tomato, *Solanum lycopersicum* [1] (old name: *Lycopersicon esculentum* Mill), originated and domesticated in Western South America and Central America from Mexico to Peru [2]. Tomato is a member of division Magnoliophyte, class Magnoliopsida, sub-class Asteridae, order Solanales, and family Solanaceae. Tomato is unquestionably the most popular garden crop worldwide [3], botanically classified as fruit (berry) but often consumed as vegetable (raw, cooked and/or in processed form), next to potato [4,5]. Although the cultivated tomato is a tropical plant, it requires moderate growing conditions and is widely adapted to different climates, thus making it cosmopolitan. China, USA, India, Turkey, Egypt, and Italy are some of the major tomato-producing countries [5]. India ranks third among the top tomato-producing countries, and it is cultivated in 6.34 million ha, with a total production of 12,433,200 tonnes and average productivity of 19,598 kg/ha [5]. The largest tomato-producing states in India include Maharashtra, Bihar, Karnataka, Uttar Pradesh, Odisha, Andhra Pradesh, Madhya Pradesh, and Assam [6]. Worldwide, more than a thousand cultivars of tomato are grown, selected based on fruit size, shapes, and growth pattern in different environments [3]. Plants of tomato are diploid (2n = 2x = 24), determinate to indeterminate growth habit, herbaceous with bisexual flowers, annual to perennial, self-pollinated, and are commonly propagated by seeds. Generally, seedlings of 4–5 weeks old are transplanted, prior to which hardening should be performed by withholding water for up to 4–5 days [7,8]. With proper water supply, tomato can be cultivated in different soil types, such as clay, black soil, and red soil; nonetheless, organic-matter-rich sandy loam soil is ideal. Tomato can also tolerate moderate saline and acidic soils. Temperature of 15–27 °C is found to be optimum for its cultivation; however, day temperatures exceeding 38 °C may adversely affect fruit set. In general, tomato is a self-pollinated crop; however, 30% cross-pollination has been recorded in some cases [8,9]. Most tomato genotypes have compound leaves, pinnately dissected with 5–9 leaflets on petioles. The inflorescence is a cyme with monochasial or dichotomous branching patterns. Flowers are bisexual and usually yellow in colour; the male part of flower is the stamens, consisting of anther borne on a filament. Anthers join laterally to form a flask-shaped cone with a prolonged sterile tip at the apex. The female flower part is the pistil, consisting of stigma, style, and ovary, and is situated in the flower’s centre, encircled by the stamens. Though tomato is commonly classified as a vegetable, it is really a fruit; berry. Most cultivated varieties, except cherry tomatoes, are either bilocular or multilocular with 4–5 locules [10]. Fruits come in a variety of shapes (globose, round, oval, flattened, elongated, or heart-shaped) and colours (golden, yellow, red, pink, purple, green, striped to white) [11]. Tomato fruits are rich in vitamin A, vitamin C, vitamin E, potassium, dietary fibre, β-carotene, and lycopene. Upon ripening, fruits develop a characteristic deep-red colour due to the pigment lycopene [12]. Among dietary carotenoids, lycopene is one of the most powerful antioxidants that protects against free radicals and is also known to prevent cancer [12]. Besides its nutritional and therapeutic properties, tomato is used for its distinct flavour and as a food colourant. Tomato fruits are also used to make a range of value-added products, such as whole-peeled tomatoes, diced products, paste, sauce, juice, and soups [13,14]. Tomato’s projected genome size is 900 megabases (Mb), containing 34,727 protein-coding genes [15]. Genes are mostly located in the euchromatin region, which accounts for less than a quarter of total genomic DNA [16]. Relatively small genome size makes tomato a genetic model for crop improvement [8] and, also, a suitable system for studying functional genomics, proteomics, and metabolomics. Further, it has been widely used for studies of various biotic and abiotic responses due to availability of various resistant genes and adaptation to a wide environmental condition and photoperiod insensitivity [17,18,19,20,21]. The tomato genome consortium sequenced the whole tomato genome as a key model plant for speeding genomic research in the *Solanaceae* family because it has the same haploid set of chromosomes and conserved genome structure (a high level of synteny) as other Solanaceous plants. Tomato was the first transgenic food (Flavr Savr Tomato) crop released for commercial cultivation in 1992 in U.S.A., genetically engineered to increase the shelf-life of fruits [22]. Being a model crop for molecular study and genetic manipulation, tomato is the highest studied crop among the vegetable crops. To date, numerous transgenes were transferred in tomato and evaluated against the biotic (virus, bacteria, fungi, and insect) and abiotic (drought, salt, heat, cold, and water logging) stress tolerance and quality improvement. Among abiotic stresses, drought is the most important stress. Drought is the inadequacy of water, which restricts tomato growth and yield [14,23]. Globally, only 14.51 percent agricultural land is well irrigated and the rest is moderately to highly drought susceptible (FAO, 2012). Drought stress primarily affects the crops by generating reactive oxygen species (ROS), which disturbs the cellular homeostasis [14,23]. In order for normal growth and development, crop must constantly adapt to climatic conditions. The water loss management in crop and detoxification of ROS are the key strategies in drought stress management. In tomato, for drought stress management, numerous transgenic tomatoes were developed. In the present study, we are providing an update of transgenic tomatoes for drought stress tolerance, which will be helpful to fulfil the research gap in tomato production under drought stress.

## 2. Drought Stress

**Figure 1:**
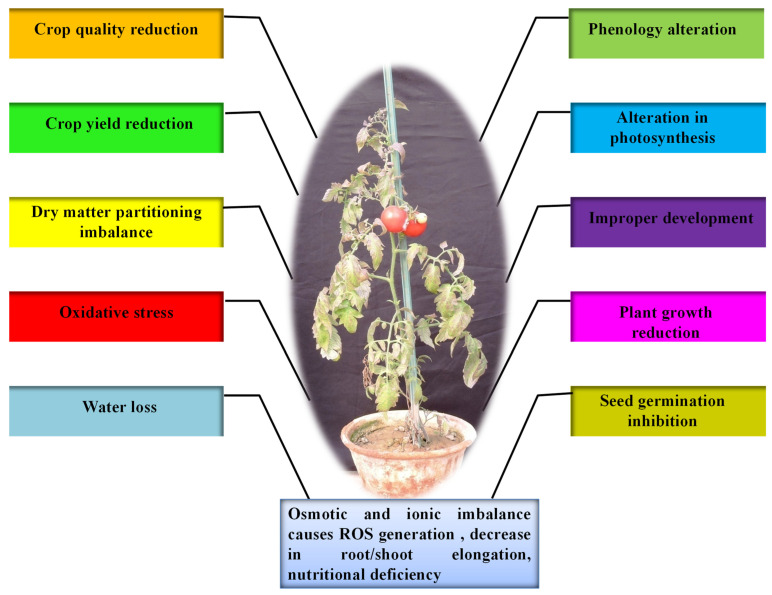
The overall impact of drought stress on tomato plant growth.

**Figure 2:**
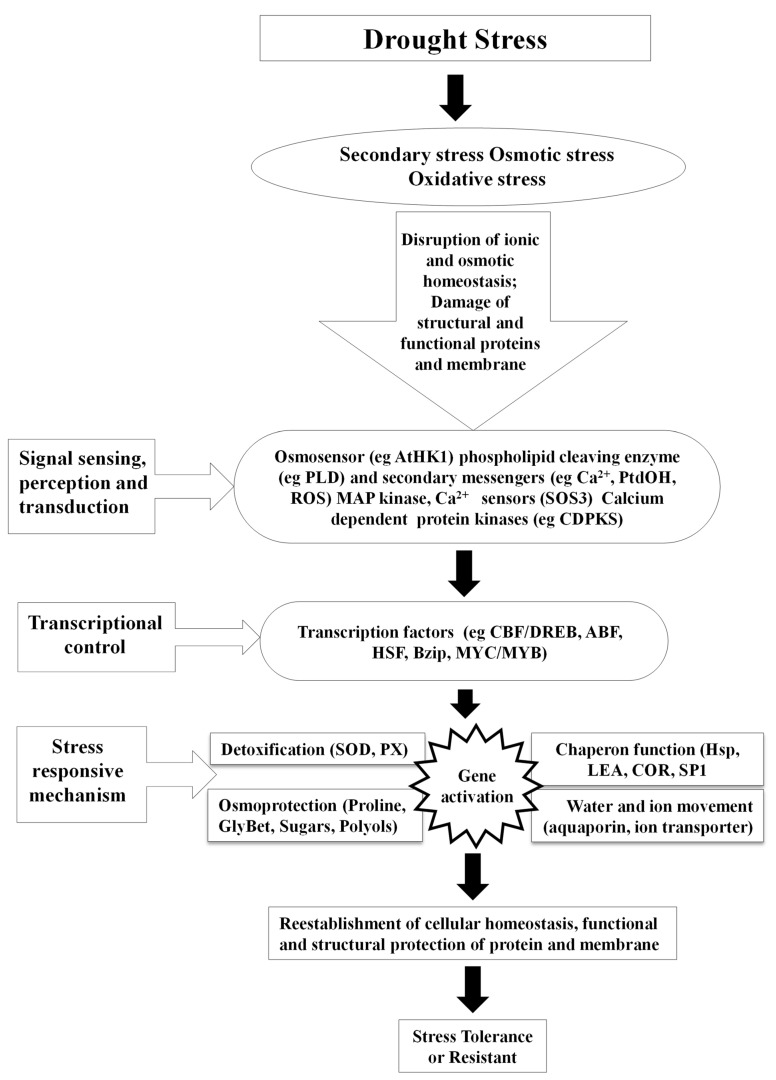
Mechanism involved in drought stress tolerance.

Any altered physiological condition that tends to disrupt plants’ homeostatic equilibrium is termed as stress [9,13,19,24,25]. Homeostasis is, in turn, a fixed value for metabolism under standard conditions, which is hardly attained by plants, as most of them are exposed to various types of stresses [24]. Drought, heat, cold, salt, high temperature and light, oxidative stress, heavy metal toxicity, radiations, and UV light are some of the constant threats to plants worldwide. The effect of biotic and abiotic factors on plants is determined by the quantity, intensity, duration, and methods of application on plants. Furthermore, plants have evolved a wide range of physiological and biochemical changes to fit and adapt to a variety of environmental challenges. Nevertheless, globally abiotic stresses are considered as the primary factor for the crop loss which roughly estimates to ~70% of yield reduction [2,24,25]. Water deficit is the most critical abiotic stress that affects many physiological parameters of plants, particularly photosynthetic capability. During drought, dehydration of the plant cells and tissues causes diminished plant growth and productivity (Figure 1). Thus, water deficit works as a limiting factor in agriculture for production by inhibiting the crop from performing its genetic yield potential [20]. Drought is characterised by inadequate availability of water, due to precipitation variability, evaporation, and soil moisture holding capacity; high water requirement by plants or a combination of all these factors restricts the plants from achieving their full genetic potential. Metrologically, drought is described as a period of below-normal precipitation that limits plant productivity in an agricultural or natural system [26]. Nearly two thirds of geographical area of India receives low (>1000 mm) or erratic rainfall. Out of 140 million hectares of cultivated area, about 68% is vulnerable to drought stress and ~34% is classified as “severe”, where the drought frequency is nearly periodic. Drought stress affects around 68% of the 140 million hectares of cultivated land, with ~34% categorised as “severe”, indicating the drought frequency being nearly periodic. Among the abiotic stresses, drought is the most severe and pervasive environmental factor [9,13,24]. Plants do expedite the detoxification and repair processes in response to stress (Figure 2), which may include alterations in the expression of ROS scavenging enzymes, *late embryogenesis abundant* (*LEA*)/*dehydrin*-type genes, and the synthesis of molecular chaperones, proteinases, and other detoxification proteins [9,27,28].

### 2.1. Drought-Responsive Transcription Factor Genes in Tomato

Drought stress causes a rise in the levels of endogenous ABA in plants [29], and a concomitant altered numerous gene expression [30] in cultivated tomato leaves. Wild relatives of *S*. *lycoperscum* are resistant to many biotic and abiotic stresses. *S*. *pennellii* (syn. *L*. *pennellii*), a wild relative of tomato, grows and reproduces with minimal water, higher water use efficiency, and resistance to wilt [31]. Martin and Thorstenson [32] used carbon isotope composition and season-long water use efficiency measures to confirm drought resistance in *S*. *pennellii*. Kahn et al. [33] discovered and isolated four tomato genes (*le4*, *le16*, *le20*, *and le25*) in response to increased levels of endogenous ABA. Kahn et al. [33] investigated the expression of these genes in *S*. *pennellii* to gain a better idea of their function during drought stress. All four ABA-sensitive *S*. *lycopersicum* genes had homologous counterparts in *S*. *Pennellii*. In *S*. *pennellii*, the increase usually occurs after a prolonged duration of drought compared to that in *S*. *lycopesocum*.

However, only three of the four genes (*le76*, *le20*, and *le2.5*) were expressed in *S*. *pennellii* in response to exogenous ABA treatment. The expression patterns of these genes in *S*. *pennellii* were usually parallel to those in *S*. *lycopersicum*, indicating a similar but undetermined function in both genotypes rather than having genes that are responsible for improved drought tolerance in *S*. *pennellii*. Members of the abscisic-acid-responsive element binding protein (AREB)/abscisic acid element binding factor (ABF) subfamily of *basic leucine zipper* (*bZIP*) transcription factors have been involved in the response to ABA and abiotic stress. In *Arabidopsis*, *AREB1* and *AREB2* functional evaluation against ABA, drought, and salinity stress conditions are revealed [34,35]; however, the tomato *AREB* function and transcriptional regulation remain unknown [36]. Yaez et al. [37] identified a basic leucine zipper (*bZIP*) transcription factor in *Solanum lycopersicum* (*SlAREB1*), *Solanum chilense* (*ScAREB1*), and *Solanum peruvianum* (*SpAREB1*). The three proteins’ deduced amino acid sequences were 97% similar and exhibited strong homology with the *ABF/AREB* subfamily of transcription factors which was demonstrated in other plant species, such as *Arabidopsis* (*ABF2*, 54% similar) and tobacco (*PHI*-2, 50% similar). The induced *SlAREB1* expression was reported in *S*. *lycopersicum* by abscisic acid, cold, and drought. For a quick investigation of genes controlled by *SlAREB1* in tomato and tobacco, a simple transient expression assay using *Agrobacterium*-mediated transformation was performed. Tobacco leaves expressing *SlAREB1* exhibited upregulation of stress-responsive genes, such as *rd29B*, *LEA ERD10B*, *TAS14*, *PHI-2*, and *trehalose-6-phosphate phosphatase* [37]. These findings imply that *bZIP* has a function in abiotic stress response in *Solanum* species. In the case of *S*. *lycopersicum*, [38] Orellana et al. identified *SlAREB1* and *SlAREB2* are induced in leaf as well as in root tissue by drought and salinity, but the effect on *SlAREB1* was more than that on *SlAREB2*. The *SlAREB1* overexpression in transgenic tomato plants improved salt and water stress tolerance compared to non-transgenic tomato plants. *SlAREB1* target gene microarray and *cDNA*-amplified fragment length polymorphism (AFLP) analyses revealed that genes coding for oxidative-stress-related proteins, lipid transfer proteins (LTPs), transcription regulators, and LEA proteins were found to be upregulated in *SlAREB1-*overexpressing tomato lines, especially in aerial tissue. The findings indicate that *bZIP* transcription factor (*SlAREB1*) is associated with ABA signal pathway that contributes to abiotic stress, as well as in pathogen response [38]. Hsieh et al. [36] found *AREB* gene, a *SlAREB* transcription factor, using a tomato *cDNA* microarray. Electrophoretic mobility shift tests revealed that the chimeric DNA-binding domain of *SlAREB* proteins can bind to the promoter regions of *Atrd29A* and *SlLAP*. However, an ABA-dependent post-translational alteration is required for the SlAREB protein trans-activation [36].

*SlAREB* constitutive expression improves tolerance to drought and salt stressors in *Arabidopsis* and tomato plants, preserving PSII, membrane integrity, and water availability in plants. Stress-related genes *AtCOR47*, *Atrd29A*, and *SlCI7*-like dehydrin were regulated by *SlAREB* overexpression in *A*. *thaliana* and tomato plants subjected to abiotic and ABA stress. Hsieh et al. [36] found that *SlAREB* regulates some genes involved in stress response and its overexpression improves plant tolerance to drought and salt stress. Islam and Wang [39] isolated full-length *LeDREB3* cDNA from tomato, investigated the pattern of expression in tomato under various abiotic stresses, and suggested that the *LeDREB3* gene may be responsible for tomato plant stress tolerance. *LeDREB3* is present in duplicate copies in the tomato genome and has considerable sequence match to DREB proteins belonging to various species of plant family. The *LeDREB3* constitutive expression reported in all the tested organs, which was especially higher in flower. Drought, low temperature, salt, and H_2_O_2_ were shown to probably enhance *LeDREB3* expression, which is expected to provide tolerance to these stresses. However, they did not evaluate its constitutive transgenic expression in tomato or any other plant species.

Investigating the ethylene response factor (ERF) protein demonstrates how it affects the expression of downstream genes in plants under stress by interacting with the GCC box, dehydration-responsive element, and C-repeat [40]. By inhibiting antisense *TERF2/LeERF2*, the ethylene-inducible ERF protein *TERF2/LeERF2* demonstrates the positive control of ethylene production in tomatoes [41]. According to Zhang and Huang [40], increased freeze tolerance in tomato and tobacco is associated with the control of *TERF2/LeERF2*, and cold gradually triggers the expression of *TERF2/LeERF2*. Similar to *Arabidopsis CBF1*–*3* genes, tomato encodes *LeCBF1*–*3* (*L*. *esculentum CBF1*–*3*), three homologs that are tandemly present within the genome [42]. However, only tomato *LeCBF1* gene is reported to be induced by cold. *LeCBF1*-3 transcripts did increase in response to mechanical agitation, similar to *Arabidopsis* CBF1-3, but not in response to ABA, drought, or high salt. *LeCBF1* is a homolog of the functional proteins *CBF1*-3 in *Arabidopsis*, and its constitutive overexpression in transgenic *Arabidopsis* increased the expression of *CBF*-targeted genes and improved tolerance to freezing. Tomato harbours an absolute *CBF* cold response pathway, according to Zhang et al. [43]. However, the tomato *CBF* regulon differs from that of *Arabidopsis* and seems to be substantially smaller and less functionally diverse. A number of dehydration-responsive elements-binding proteins (*DREBs*) in plants have been identified, and it has been suggested that both abiotic and biotic stressors can activate them. Recently, Guo and Wang [44] isolated a *DREB* gene from tomato and designated it as *LeDREB2*.

*LeDREB2* is classified in *DREB* family with the *AP2* group. Being a single-copy gene of the tomato genome, it expresses strongly in young leaves and roots but shows weak expression in shoots and mature leaves. Various environmental stresses, such as cold and draught, induce the transcription of *LeDREB2*. Different studies demonstrate the expression analysis inducing the transcription of *LeDREB2* gene due to various stresses (H_2_O_2_, salt, ABA, and methyl viologen) in tomato. Guo and Wang [44] found that *LeDREB2* gene is a *DREB* transcription factor involved with oxidative and abiotic stress in tomato. Recently, Li et al. [45] cloned *SlDREB* from cultivated tomato *M82*, a transcription factor, and found that it plays a negative role in architecture of tomato plant, and elevates tolerance against drought. Expression profiles indicate that *SlDREB* expression is mainly expressed in leaf and stem and is unable to induce by abscisic acid (ABA) but is suppressed by ethylene and GA. The activity of yeast assay exhibited that *SlDREB* exclusively binds to *dehydration responsive element/C-repeat* (*DRE*/*CRT*) of the *SlCPS* promoter region. When constitutively overexpressed, the *SlDREB* altered plant morphology by regulating leaf and internode elongation, and the consequent dwarfism of tomato plants could be reversed by adding gibberellic acid (GA3) exogenously. The findings of Hsieh et al. [36] showed constitutive overexpression of *SlAREB* in tomato induced dwarfism that could be alleviated by exogenous *GA3* treatment, which were comparable to findings of [45]. Transcript level analysis of transgenic plants disclosed that *SlDREB* overexpression resulted in dwarf phenotype by downregulating key *GA* biosynthesis genes involved, such as *ent*-copalyldiphosphate synthase (*SlCPS*) and GA 20-oxidases (*SlGA20ox1*, -*2*, and -*4*), thereby reducing endogenous GA levels of application in transgenic plants.

### 2.2. Genetic Engineering of Tomato for Improved Drought Stress Tolerance

In the last century, efforts through classical breeding have resulted in improved agronomic traits, as well as nutritional value of cultivated tomato. However, traditional breeding has not been very successful in enhancing drought tolerance in tomato cultivars due to the limited genetic variation within *S*. *lycopersicum* species. It is evaluated that just ~5% of the complete genetic variability inside the tomato family can be found within *S*. *lycopersicum* (100 Tomato Genome Sequencing Consortium, 2014). The genes related to different required agricultural traits, including drought resistance, do not exist in this cultivated species [13]. However, fortunately, related wild tomato species, including S. *pimpinellifolium*, *S*. *chilense*, *S*. *peruvianum*, *S*. *pennellii*, and *S*. *hirsutum*, have shown to be a rich source of the genes and traits needed to increase resistance to various abiotic stresses. Due to the intricacy of abiotic stress tolerance mechanisms, transferring these traits from wild relatives of *S*. *lycopersicum* is challenging and takes a lot of effort; therefore, they have not been fully utilised [13]. The genetic base for resistance and tolerance against abiotic stress in species of wild tomato are hereditary as quantitative traits; thus, it is also unlikely that a single gene from wild tomato species expressed in cultivated species will confer drought tolerance [46]. For such reasons, developing stress-tolerant transgenic tomato taking regulatory genes from related or distant species is a workable approach to improve drought tolerance of tomato. Therefore, genetic engineering is a relatively quick, precise, and frequently successful method of improving abiotic stress resistance in many plant species.

Considering the problem of drought stress in tomato, attempts have been made to enhance drought tolerance by incorporating single genes from distant plant species [8,19,20,24,27,47], as well as some microbes, governing drought and other water-deficit stress tolerance. Constitutive overexpression of *Arabidopsis CBF1* in tomato brought about upgraded plant resistance to cold, dry season, and salt burdens. In any case, this upgraded resistance included some significant pitfalls, causing decreased plant development and yield [19]. The transgenic tomatoes showed improved resilience to dry season, cold, and salt stress when a related gene (*CBF1*) was expressed using an ABA/stress-inducible *ARBC1* promoter from the barley *HAV22* gene. Additionally, the use of the inducible promoter eliminated the negative effects of the ectopic expression of *CBF1* on plant growth and yield [19,27]. By ensuring various proteins, boiling stable proteins (BSP), have been involved in parching resistance against drought stress. Drought stress resistance of transformed tomato plants was attempted with a special 66-kD BSP from *Populus tremula* using polyethylene glycol (PEG) study, biomass investigation, proline assay, and electrolyte leakage measurement. These plants displayed slightly increased drought stress tolerance [48]. *S*. *lycopersicum* was found to contain the basic leucine zipper (*bZIP*) transcription factor *SlAREB1* [37]. Uncomplicated transitory expression analysis was implemented for fast study of genes controlled by *SlAREB1* in tobacco and tomato recommended regarding the group of *bZIP* that performs a role in abiotic stress reaction in the *Solanum* genus. Zhang et al. [49] developed marker-free selectable transgenic tomato plants exhibiting increased resistance to drought, cold, and oxidative stresses, which constitutively expressed *AtIpk2b*, an inositol polyphosphate 6-/3-kinase gene from *A*. *thaliana*. Tobacco *osmotin* gene driven by *CaMV35S* promoter was transferred to tomato and physiological analysis at T_1_ and T_2_ generations was used to test for resistance against drought and salt stress. Increased resistance to drought and salt stress was observed during NaCl stress and when water was withheld [47]. Tomato plants were transformed with the *mannitol-1-phosphate dehydrogenase* (mtlD; EC1.1.1.17) gene from bacteria, which is controlled by the *CaMV35S* promoter. Drought, cold, and salinity resistance study showed that transgenic lines perform a better resistance against abiotic stresses [50]. The *SlAREB1-*overexpressing transgenic tomato plants exhibited increased resistance against drought and salt stress, as analysed by various physiological parameters, such as relative water content, damage by lipoperoxidation, and chlorophyll fluorescence [38].

By offering the codA gene expressing a choline oxidase from *Arthrobacter globiformis*, Goel et al. [51] modified tomato for increased resistance to salt and water stress with the capacity to assimilate glycine betaine. The *codA* transgenic plants demonstrated increased resistance to salt stress during seed germination and ensuing development of young seedlings compared to wild varieties of plants. Developed *codA* transgenic plants had uncovered more elevated levels of relative water content, chlorophyll concentration, and proline level. Rai et al. [19,24] developed transgenic tomato *cv*. Kashi Vishesh (H-86) for improved drought tolerance by using the *BcZAT12* gene coding sequence regulated through *Bclea1a* promoter. *BcZAT12* expresses a C_2_H_2_ type zinc finger protein that is known to confer multiple abiotic stress tolerance to plants. Analysis of RWC, EL, CCI, H_2_O_2_ and superoxide anion level, and antioxidant enzyme activities suggested that *BcZAT12* tomato transformants had increased levels of drought tolerance. Transgenic plants with the *A*. *thaliana* transcription factor *ATHB-7* gene inserted displayed reduced stomatal density and increased tolerance to drought stress [52]. However, *AtATHB*-7 was established to be expressed increasingly under drought stress; thus, it performs as a negative growth regulator. The integration of drought-stress-tolerant genes from unrelated species into tomato has been the focus of intense research efforts from scientists all over the world (Table 1) [47,48,49,50,51,52,53,54].

#### 2.2.1. *DREB1A*/*CBF3* Gene in Stress Tolerance

Tolerance to abiotic stress in *Arabidopsis* [72,73], maize [74], and rice [75] is often achieved by modifying endogenous plant pathways, frequently by manipulating the overexpression of important regulatory proteins or transcription factors. Numerous transcription factors are part of large families [13,30]. Manipulating abiotic stress tolerance traits is complicated, as the interaction of plants with the environment is a continuous and intricate process. A large number of TFs are present in the plant genome. About 5.9% of *Arabidopsis* genome is devoted to coding more than 1500 TFs. On the basis of the composition of their binding domains, these TFs may be divided into a number of families [76]. Many of these TFs, including *MYB*, *AP2/EREBP*, *bZIP*, and *WRKY*, are members of large multi-gene families. However, certain stress-sensitive genes may share the same TFs, as demonstrated by the significant overlap of the gene expression profiles that are activated in response to diverse stress stimuli. Individual members of the same family may respond differentially to distinct stress stimuli [19,21,24,28].

The transcription factors (TFs) associated with the drought and cold stress tolerance are *ABA*-triggered and come from a variety of classes, including the *bZIP*, *Zinc finger*, *homeodomain Leu zipper (or HD-Zip)*, *MYC/MYB*, and *ABI3/VP1* families [24]. The *bZIP* TFs form dimers with *ABA response elements* (*ABREs*); however, a second “cis element” or “coupling element” is often needed for optimum ABA responsiveness [1]. Sometimes this CE element and the dehydration-responsive element (*DRE/CRT*) are comparable [27]. According to [77], the *DRE/CRT*-binding *AP2s* and the *ABRE*-binding *bZIPs* may interact to regulate the ABA-regulated gene expression. The *CRT* binding factors are one class of *AP2* TFs that are essential in both the ABA-dependent and ABA-independent pathways (*DREB1s* or *CBFs*; [20]). In *Arabidopsis*, cold induction *CBF1*-3 genes are ABA-independent; however, ABA is required for the dehydration-induced activation of the *CBF4* gene [21]. The expression of all *CBF* genes in *Arabidopsis* is low under normal growth conditions, but it quickly rises in response to water stress [78].

There are regulators of the *CBF* genes also: [79] identified *ICE1* in *Arabidopsis*, a *bHLH* TF, which specifically binds to *MYC* detection sequences within *CBF3* promoter and triggers expression of *CBF3* in the cold. *SFR6* could collaborate with the *CBFs* or *DREB2s*, as shown by the *Arabidopsis sfr6* mutant’s lack of *COR* gene expression that is controlled by *CRT*/*DRE* in response to osmotic stress, cold, or exogenous ABA administration [80]. Quite the opposite to the *ICE1* and *SFR6*, the *HOS1* is identified as a negative regulator of *CBF2* and *CBF3* that encodes for a RING finger protein, which is capable of work in ubiquitin-based nuclear protein breakdown and can cause increase in ICE proteins turnover [81] (Sarkar et al., 2019). The *RAV1* (AP2; Lehti-Shiu et al., [82]) and *ZAT12* (Zn finger; Rai et al., [24]) have similar expression patterns to those of *CBF1*–*3*, and are known to operate in parallel pathways to those of the *CBFs* [27]. The *DREB2* and *AP2*, two other transcription factors, play a role in adaptation to drought in an ABA-independent mode [83]. In contrast, RAP2.6 and RAP2.1, which are AP2 domain proteins, induced only after the initial inductions of *CBF1*–*3*, *RAV1*, and *ZAT12*, indicating that these are downstream TFs [84]. Medina et al. [85] indicated that the *RAP2*.*1* might be a target of the *CBF1*–*3* activators, and *RAP2*.*1* promoter sequence consists of two copies of the CRT/DRE core sequences. Plants’ drought adaptation mechanism is heavily regulated by the transcription process [42].

Therefore, one of the ideal targets to design plants for better drought tolerance is such transcription factor genes that regulate the drought adaptation process. However, not every TF engaged in signal transduction of drought is an appropriate target for deployment in transgenics. Some proteins require post-translational modification for its function in transgenic plants [86]. For instance, despite the possibility that the *DREB2* TFs play a crucial role in the gene expression that is controlled by drought, the overexpression of *DREB2 cDNAs* in transgenic plants only leads to poor induction of the downstream genes and does not result in drought tolerance [58]. The *AP2/EREBP* family of transcription factors that can bind to the *DRE* element was discovered, and these factors were given the names *CBF1/DREB1B*, *CBF2/DREB1C*, and *CBF3/DREB1A* [83,85]. To develop abiotic stress tolerance, many plant species have been successfully transformed using *DREB*/*CBF* genes. The orthologs of *CBF* genes are present in most crop plants, including apple, canola, tomato, rye, strawberry, barley, rice, wheat, corn, sweet cherry, ryegrass, chrysanthemum, and broccoli [86,87,88,89,90,91,92,93,94,95,96,97].

Functional analyses of a number of putative orthologs have helped to identify the pathway for conservation in those plant species. *Arabidopsis* [98], tomato [19], chrysanthemum [99], peanut, and many other plant species have increased drought tolerance when the *Arabidopsis DREB/CBF* genes are expressed constitutively. Similar to this, constitutive overexpression of the rice ortholog CBF (*OsDREB1*) in *Arabidopsis* demonstrated tolerance to salt, cold, and drought [90]. Similarly, constitutive *ZmCBF* gene overexpression in maize also showed enhanced cold tolerance. It was noted that *DREB*/*CBF* constitutive expression effected increase in stress tolerance, but the ectopic overexpression of *CBF* genes produced dark-green and dwarf plants [53,54]. Stress-inducible promoters have been combined with the *DREB*/*CBF* genes to solve these issues [18,19,100]. These promoters’ expression is very low under normal growth condition and achieves enhanced stress tolerance without retarding growth characteristics.

Stress can induce the *CBF/DREB1* family members *CBF1*, *CBF2*, and *CBF3* (or *DREB1B*, *DREB1C*, and *DREB1A*). The *AtCBF1*-3 genes are arranged in a direct repeat on *Arabidopsis* chromosome 4. *Cor6*.*6*, *Cor15a*, *Erd10*, *Kin1*, *Kin2*, *Rd29*, *Rd17*, and other genes are arbitrated by *DREB*/*CBF* proteins, which are encoded by the *AP2/EREBP* multi-gene complex. The promoter region of *CBF* genes does not contain the *DRE* sequence; therefore, *CBFs* are not subjected to auto-regulation. In *Arabidopsis*, the expression levels of *CBF* increases with several minutes of stress exposure, followed by accumulation of *COR* and dehydration-responsive genes at about 2 h [101].

Single TF overexpression in transgenic *Arabidopsis* plants may have improved stress tolerance. When *Arabidopsis* was transformed with the *DREB1A* gene [102], cauliflower mosaic virus (*CaMV35S*), or by a *DRE*-containing promoter from the dehydration-induced genes (*rd29A*), it displayed a distinctly increased tolerance to drought, freezing, and salt stress. Additionally, *CBF3* overexpression in *Arabidopsis* improved resistance to freezing and, more intriguingly, demonstrated numerous biochemical changes related to acclimatisation to the cold, including increased proline and total soluble salt levels [98]. It is clear from the elevated *P5CS* transcript level found in plants overexpressing *CBF3* that the increase in proline levels is the result of increased expression of a crucial proline biosynthesis enzyme.

#### 2.2.2. ZFP (ZAT) Gene in Stress Tolerance

Several plant (*Arabidopsis*) genomic and *cDNA* clones encoding potential *ZFPs* were isolated as a result of knowledge of the *C_2_H_2_* zinc finger protein (*ZFP*) gene family, the largest group of regulatory proteins crucial for growth and development in animals [103,104,105]. Today several classes of *ZFPs* are reported in plants, which are either single-finger C_2_H_2_ ZFP gene, as in *Arabidopsis* [106], or two-fingered *C_2_H_2_ ZFP* gene in *petunia* and wheat [107,108], or three-fingered *C_2_H_2_ ZFP* gene in *Arabidopsis* [103], or the ZFPs which are not C_2_H_2_ type [109]. Sequence comparison of these different C_2_H_2_-ZFPs in *Arabidopsis* showed that the first finger always contained the amino acid motif (F/Y) QALGGH [103]. Moreover, studies have revealed that C_2_H_2_-type *ZFP* gene overexpression caused both the activation of stress-responsive genes and increased salt tolerance, dehydration, cold stress and/or heat stress [110,111,112,113].

Meissner and Michael [103] were the first to isolate and characterise a diverse family of *C_2_H_2_*-type zinc finger proteins in *Arabidopsis*. These workers used a PCR approach for the ZFPs with an encoding potential and reported that there existed two- as well three-fingered plant C_2_H_2_ ZFP having an evolutionary origin. The PCR products of *ZFP* gene resulted in eight *ZAT1*, *ZAT2*, *ZAT* (3, 4, and 5), *ZAT7*, *ZAT8*, and *ZAT9* clones. Further search of the database using *Pszf1* as reference for the potential *C_2_H_2_*-*ZFP* clones resulted in one cDNA corresponding to *ZAT12* and two new clones, which were named by these workers as *ZAT10* and *ZAT11* [103]. The *ZAT10* and *ZAT12* cDNA sequences had an absence of upstream in-frame stop codons, suggesting that they are different from the other *ZAT* genes. There is also a possibility that there is N-terminal extension to the open reading frame to *ZAT10* and *ZAT12* genes. Similar genomic clones were also obtained and, by comparison of the genomic sequences and cDNA clones, these researchers reported the absence of introns in the gene. *ZAT10* and *ZAT12* genes were widely expressed when mRNA transcripts were studied, specifically under exposure to light stress, suggesting that they are stress inducible. The comparison of ZFPs (*ZAT12*) from *Arabidopsis* showed two main families characterised by TF *EPF2* and *EPF1*.

Several members of the zinc-finger protein (*ZFP*) family, such as *ZAT7*, *ZAT10*, and *ZAT12*, respond to a variety of abiotic and biotic stimuli, despite the fact that many signalling and regulatory genes are believed to be stress-specific [110,111]. In *Arabidopsis*, *ZAT12* is crucial for abiotic stress and reactive oxygen signalling [110]. Davletoava et al. [110] also verified that stimulation of *ZAT12* expression under various abiotic conditions is activated at the transcriptional level when using a fusion between the *ZAT12* promoter and *luciferase* gene.

Using *ZAT12* gain- and loss-of-function lines, these workers confirmed *ZAT12* function during high light and heat stresses, oxidative, osmotic, and salt stress. Transcriptional profiling of *ZAT12*-overexpressing transgenic and wild-type plants subjected to H_2_O_2_ stress showed that, in *Arabidopsis*, *ZAT12* constitutive expression results in the increased oxidative and light-stress-responsive transcript expression [114]. Therefore, *ZAT12* under specific growth conditions accelerated to govern transcript accumulation involved in response to high light and oxidative stress in *Arabidopsis*. These researchers validated *ZAT12* function under conditions of high light, heat, oxidative, osmotic, and salt stress using *ZAT12* gain- and loss-of-function lines. Transcriptional profiling of *ZAT12*-overexpressing transgenic and wild-type plants overexpressing *ZAT12* in response to H_2_O_2_ stress showed increased expression of oxidative- and light-stress-responsive genes in *Arabidopsis ZAT12* [114]. *ZAT12* thereby increased transcript accumulation in response to high light and oxidative stress in *Arabidopsis* under specific growth conditions.

According to functional study of a *C_2_H_2_*-type zinc finger protein, the *ZFP179* (*ZAT12*) protein has implications for various cellular processes involved in plant growth and stress responses in rice [111]. These researchers used transgenic rice to examine how well it tolerated oxidative stress, salt stress, drought stress, and auxin treatment. They found that the transgenic line carrying a transgene from the *ZFP179* family displayed significantly increased oxidative stress tolerance, ROS scavenging ability, and expression levels of several stress-related genes, including *OsDREAB2A*, *OSP5CS*, *OsProT*, and *Oshea3*, under salt stress.

In the transgenics, proline and soluble sugars accumulated as well, according to Sun et al. [111], indicating that the rice *ZFP179* promoter is sensitive to salt stress. Rai et al. [24,27] showed that, under drought stress, the ectopic overexpression of *BcZAT12* gene in tomato had low electrolyte leakage, high relative water content, chlorophyll and antioxidative enzymes (APX, CAT, SOD, GR, and POD) activity, and, also, the lower accumulation of ROS. The upregulation of the drought-responsive *ZAT10* gene was shown by transcriptome analysis in drought-tolerant transgenic plants overexpressing *TF ABF3* from *Arabidopsis* [115]. Additionally, the drought response did not activate an unexpected gene network (pleiotropic effects).

#### 2.2.3. Multigenic Transgenic Approach for Abiotic Stress Management

Both biotic (virus, bacteria, fungi, and insect) and abiotic (drought, salt, heat, cold, and water logging) stresses cause direct as well as indirect damages to plant growth and development by affecting various molecular pathways. These pathways are regulated by a cascade of numerous genes and, hence, targeting a single gene may not solve the issue. Transgenic crops with stacked traits are those which have multiple characters within the same plant conferred by multiple gene introduction with the help of genetic engineering. In 2018, genetically modified crops with a combined trait occupied about 42% global biotech crop cultivation area, most commonly in the United States and Brazil (ISAAA, 2019). For example, stacked soybean event, MON87701 × MON89788 (trade named Intacta™ Roundup Ready™ 2 Pro), harbours *cp4 epsps* and *cry1Ac* genes, respectively, for glyphosate herbicide and Lepidopteran resistance. Furthermore, many crop plants have been developed by stacking multiple transgenes for harbouring resistance against various insect pests and/or herbicide tolerance by various mechanisms. The multiple transgenic stacked maize, event MON-89-34 × DAS-15-7 × MON-88-17 × DAS-59122 (trade name Genuity^®^ SmartStaxTM), carries the *cp4 epsps* and *pat* genes for herbicide glyphosate and glufosinate-ammonium tolerance, as well as the *cry1Fa2*, *cry2Ab2*, *cry1A*.*105* and *cry35Ab1* genes, respectively, for Lepidopteran and Coleopteran resistance (ISAAA, 2019). Nevertheless, number of molecular pathways and important gene(s) involved in abiotic stresses from different plants were identified and used in the development of double, triple and/or multitransgenic plants [116,117,118,119,120,121,122]. To cope with multiple abiotic stresses, multigenic transgenic crops, such as rice, cotton, soybean, sweet potato, tobacco, tomato, sugarcane, etc., have been developed [123,124,125,126,127]. For the first time, Zhao et al. [123] coexpressed the *SsNHX1* and *APV1* gene, respectively, from *Suaeda salsa* and *Arabidopsis* in rice for salt stress tolerance. They reported a greater salt tolerance in *SsNHX1* and *APV1* gene co-expressing three-week-old rice seedling than *SsNHX1* gene alone in 300 mM NaCl in field growth condition and 150 mM NaCl concentration in MS medium. They also reported increased photosynthesis and root proton exportation capability and reduced H_2_O_2_ formation under salt stress in *SsNHX1* and *APV1*-gene-co-expressing seedlings. After this, many genes from different sources were co-expressed in various important crops to manage different abiotic stresses (Table 1). Bhaskaran and Savithramma [117] developed the double transgenic tomato plants co-expressing *PgNHX1* and *AtAVP1*, respectively, from *Pennisetum glaucum* and *Arabidopsis thaliana* for salt stress tolerance. The tomato plants harbouring both *PgNHX1* and *AtAVP1* gene exhibited greater salt tolerance than *PgNHX1* and *AtAVP1* single-gene transgenic plants. They reported that, at 200 mM NaCl concentration, *PgNHX1* and *AtAVP1* co-expressing plants grow well, while single transgenic and wild-type plants showed chlorosis and fail to survive within three weeks. The transgenic tomato lines harbouring *PgNHX1* and *AtAVP1* genes showed higher chlorophyll and proline accumulation 1.4 times higher under salt stress compared to single-gene transformants. Furthermore, the double transgenic plants showed 1.5 times higher accumulation of Na^+^ content in their leaf compared to single-gene transformants. The *PgNHX1* and *AVP1* co-expression enhanced the osmoregulatory capability of double transgenic lines by enhancing ion sequestration into the vacuole by increasing the proton availability and, thus, reducing the Na+ toxic effect. Recently, Viveros et al. [118] co-expressed the *BjGlyI* and *PgGlyII* genes in tomato, cv. Ailsa Craig, respectively, from *Brassica juncea* and *Pennisetum glaucum* to enhance salt tolerance. The *GlyI* and *GlyII* overexpressing double transgenic lines under 800 mM NaCl concentration showed decreased H_2_O_2_ accumulation and lipid peroxidation in leaf tissues. They also reported a significant decrease in the chlorophyll a and b content in wild-type (WT) than the line co-overexpressing *GlyI* and *GlyII*. The *GlyI* and *GlyII* gene are a component of the glyoxalase system, which plays a significant role in different physiological processes and enhances abiotic stress tolerance, including salt, by reducing oxidative damage. Baghour et al. [128] developed *LeNHX2* and *SlSOS2* co-overexpressing tomato, cv. MicroTom, respectively, from *Solanum esculentum* and *Solanum lycopersicum* to increase salt tolerance and production. The double transgenic tomato co-overexpressing *LeNHX2* and *SlSOS2* exhibited greater salt tolerance than *LeNHX2-* or *SlSOS2*-expressing tomato lines at 120 mM NaCl concentration in hydroponics and, also, in pot experiment with successive salt irrigation (120 mL of 75 mM NaCl thrice per week for eight weeks). They observed enhanced physicochemical performance of double transgenic tomato lines under salt stress. Earlier, we pyramided [14,23] *AtDREB1A* and *BcZAT12* gene under the control of ectopic promoter *rd29* and *lea1*, respectively. We noted that the tomato lines independently expressing *AtDREB1A* and *BcZAT12* genes showed higher drought stress tolerance compared to the tomato expressing individual genes, either *AtDREB1A* or *BcZAT12*. The findings of Krishna et al. [14,23] also suggest that the multigenic transgenic tomato can be a potential tool against the drought stress as they regulate different regulatory pathways under drought stress.

#### 2.2.4. Genome Editing in Tomato for Drought Stress Tolerance

Recently, the development of clustered regularly interspaced short palindromic repeats (CRISPR)/CRISPR-associated protein (Cas) (CRISPR/Cas9)-mediated genome editing opened the new avenue of vegetable improvement for virus resistance. CRISPR–Cas9 is the most studied system, and the endonucleolytic complex (Cas9-guide RNA (gRNA)-tracer RNA (tracRNA) is programmable for the site-specific dsDNA cleavage. Any target in the DNA sequence followed by NGG (the protospacer adjacent motif, PAM) can be cleaved by Cas9–sgRNA (engineered single-guide RNA) complex [129,130]. The 20 nt sequence of the sgRNA complementary to the target DNA works as a guide and the stem-loop scaffold binds and activates Cas9 for sequence-specific nuclease activity. The CRISPR/Cas9 edits the host tomato plant gene(s) and provides molecular immunity against the drought stress. In host tomato plant, there are many genes reported which positively regulate drought stress tolerance by encoding protein(s), which facilitates [130,131,132] edited tomato *SlNPR1* gene via CRISPR/Cas9 and reported a higher drought stress tolerance in *SlNPR1* edited lines. The *SlNPR1* gene encodes for nonexpressor of pathogenesis-related protein 1, its regulatory mechanisms are well studied in the case of biotic stresses; however, in case of drought stress, it is still unexplored. Recently, Liu et al. [130] edited tomato *SlLBD40* gene and found that the mutants were displaying drought tolerance even after stopping irrigation for 10 days. Tomato *SlLBD40* is a plant-specific transcription factor, which regulates stomatal conductance. The mutation in *SlLBD40* reduces the stomatal conductance. Earlier, Chen et al. [132] mutated tomato *SlARF4* gene, a gene encoding auxin response factors using CRISPR/Cas9 and they reported that mutants were showing higher drought tolerance after stopping irrigation for 12 days. The tomato *SlARF4* plays important roles in different physiological processes of tomato plants, although the exact function of *SlARF4* still unknown under drought stress. The genome editing in tomato is underway for the development of drought stress tolerance and there are very few reports available on tomato. Therefore, there is need for the identification of tomato gene(s) and their pathway for drought stress tolerance. The identification of gene(s) and pathways will facilitate the multigene editing for drought stress tolerance.

## 3. Yield Potential of Transgenic Tomato under Drought Stress

Being frequent and uncertain, drought stress potentially reduces the tomato yield and quality. To fulfil global tomato demands and ensure tomato fruit quality, transgenic technology is applied. The ultimate goal of introducing transgenic technology is to maintain genetically predetermined yield and quality in tomatoes. The development of fruits in tomato is itself a very complex biological process and governed by genes of auxins, gibberellins, and ethylene and the yield of tomato mainly depends on the expression of *OVATE*, *SUN*, *EJ2*, *CNR*, *SlKLUH*, and *CSR* genes, which regulate fruit shape and weight [133]. Drought reduces yield in tomato by delaying physiological processes; to overcome this, a number of transgenes have been introduced in tomato having different modes of action, such as ROS detoxification, osmoprotective, water use efficiency, water loss reduction, etc. [13]. In 2003, *AtCBF1* regulated by ABA/stress-inducible promoter was transformed in tomato by [134] to discover the yield potentiality and survival under drought stress. After four-week exposure to drought by withholding irrigation, they found that transgenic tomato lines viz. AC1, AC2, and AC3 yielded 102.2, 115.0, and 101.0 g per plant, respectively, while, under the same condition, wild type yielded only 32.0 g. Furthermore, transgenic tomato lines also excellently survived compared to wild type AC1, 25; AC2, 28; AC3, 29; and wild type, 0 plants out of 30 plants. The *CBF1* is a transcription factor that regulates multiple molecular pathways under drought stress to modulate the drought and improve plant growth and development. Tomato variety Yaxin 87−5 was transformed by using *Christolea crassifolia HARDY*, a *AP2/ERF*-like tanscritpion factor (*HARD* gene), by Guo et al. [65]; they reported that the transgenic tomato lines expressing HARDY have better plant growth attributes and yielded 38.65–44.65% higher compared to the wild type, respectively, after 16 days of drought stress. Wang et al. [135] developed transgenic tomato by using MdPIP1;3 an aquaporin gene from Malus domesticus, and they evaluated yield potential of transgenic tomato under artificially induced drought by stopping irrigation for 20 days (control 43–45% soil moisture content and drought 0.5 to 1%) and reirrigated to maintain 43–45% soil moisture. They reported that the fruits of transgenic tomatoes are heavier and larger than wild type and the yield was approximately 1.2% higher under drought stress than wild type. They also reported that 77% of transgenic lines’ tomato stomata became closed, while, in wild type, 65% stomata remained open. Thus, transgenic tomato managed drought stress and the aquaporin translocated more water in fruits during developmental stages. Improving water use efficiency in tomato is considered as a potential tool to cope with drought stress. Kelly et al. [136] transformed tomato (cv. MP-1) with *A. thaliana* guard-cell hexokinase (*AtHXK1*) and they reported that the expression of *AtHXK1* in tomato gourd cell improved water use efficiency by 10–20% under drought stress. Earlier, we [14] evaluated transgenic tomato expressing *AtDREB1A* and *BcZAT12* individually and in pyramided form (*AtDREB1AXBcZAT12*) for yield potential under drought stress (0, 7, 14, and 21 days of irrigation withholding; soil moisture contents were 80, 40, 25, and 15% of the field capacity, respectively). We reported that the increasing intensity of drought potentially reduced the tomato yield in all the transgenic lines and wild type, but the decrease was high in wild type. Under 25% moisture content *AtDREB1A* and *BcZAT12* transgenic tomato lines yielded approximately 1.8 times, and *AtDREB1AXBcZAT12* lines approximately 3 times higher yield than wild type at similar moisture content. This higher yield in *AtDREB1AXBcZAT12* lines were probably due to independent mechanisms of molecular pathway regulation by *AtDREB1A* and *BcZAT12* genes. Thus, the transgenic tomato plants have yield potential under drought stress and can play an important role in water management and fulfilling tomato needs. Furthermore, the transgenic tomatoes must be scientifically evaluated for bio and environment safety to resolve social and ethical issues related to the transgenic tomatoes.

## 4. Conclusions and Future Prospects

Due to climate change, the frequency of uncertain and uneven rain fall increasing over time, this phenomenon will make drought very severe in coming times. Drought stress affects almost every crop, including tomato. Being a plant model system, tomatoes are extensively utilized to understand plant biological processes, such as functional genomics, proteomics, and metabolomics. In recent years, extensive work has been carried out in tomato at the molecular level to cope with drought stress and numerous transgenic tomatoes developed by utilizing microbial and nonhost plant gene(s). Recently developed CRISPR/Cas9-mediated genome editing in tomato avoids introduction of transgene and can edit the host plant genome for specific gene(s) up- or downregulation for the drought stress tolerance; hence, it is safer than transgenic technology. The CRISPR–Cas9-mediated genome editing for drought stress tolerance will insure ecologically and economically friendly quality tomato production under drought stress and will be helpful for food and nutritional security.

In future, to fulfil continuous tomato fruit demand, there is need for modification of the tomato genome through transgenic technology and genome editing approaches. It is well established that manipulation of single or multiple genes in tomato improved the drought stress tolerance. Furthermore, targeting drought-stress-related gene(s) will not be enough to ensure sustainable tomato production, as other abiotic stresses, such as salt, heat, and cold, also share water scarcity due to different physiological phenomena. Therefore, multiple abiotic stresses should be targeted to ensure tomato survival under adverse environmental conditions and give potential yield. There is also a need for better understanding about the different molecular, biochemical, and metabolite(s) identification involved in different abiotic stresses. A deeper understanding of stress tolerance mechanism in tomato by metabolomics is required, which could open new avenues of metabolic pathway manipulation to develop abiotic stress tolerance in tomato. The manipulation of metabolic pathways is quite difficult due to the majority of proteins of a pathway interacting with the proteins of other pathways. Therefore, metabolic pathway manipulation will only be possible by controlling multiple genes of the same pathway or other interlinked pathways. Transgenic crops have been cultivated in United States of America for more than two decades, and one decade in India, without any reported harmful impact on human beings and the environment but the path of new genetically modified tomatoes to a farmer’s field is not smooth. The transgenic tomatoes have survival and yield potential under drought stress but their global acceptance is under question at the global level because of social, ethical, and political issues. Therefore, there is need for in-depth understanding about science, mode of action behind the transgenes, and all the possible studies related to the safety of transgenes and their harmlessness. Hence, there is urgent need to raise awareness among the farmers and citizens about the benefits of transgenic crops on a scientific basis. It also important to develop understanding among the public that the transgenic crops are safe for both human beings and the environment with the help of facts and biosafety data recorded on transgenic crops. The commercial release of *Bt* brinjal in Bangladesh is an excellent example of tackling such hurdles successfully by inviting a representative of the opposing organisations at every developmental stage of *Bt* brinjal and scientific clarification of their doubts. Such type of activities would raise awareness about the safety process evaluation of transgenic crops. The scientific document should bring in the public domain, answering each and every doubt of opponents and farmers in an easy form. Finally, great efforts are needed for harvesting the fruits of this wonderful technology in coming time.

## Figures and Tables

**Table 1:** Transgenes used for development of drought stress tolerance, their function, and mechanism of action.

S. No.	Gene	Function	Mechanism of Action	Reference
1	*CBF1*	Stress-inducible transcription factors	Expression of stress-responsive gene	[55]
2	*H1-S*, *drought induced linker histone*	DNA packaging and organisation of chromosomes in the nucleus	Modulation of mechanisms related to the stomatal function	[56]
3	*AtCBF-1*	Stress-inducible transcription factors	Expression of stress-responsive gene	[57]
4	*H* *+* *-pyrophosphatase*	Facilitate auxin fluxes	Enhance pyrophosphate-driven cation transport into root vacuolar fractions	[58]
5	*bspA*	Protein protection	Enhance desiccation tolerance by protecting proteins in membranes and cytosol	[48]
6	*coda*	Accumulation of glycine betaine	Osmolyte accumulation to protect against oxidative damage.	[59]
7	*LeNCED1*	Increase in abscisic acid (ABA) accumulation	Stomatal closure and increased water-use efficiency (WUE)	[59]
8	*Osmotin*	Stress-responsive multifunctional protein	Osmotin provides protection via different mechanisms related with programmed cell death	[47]
9	*PtADC*	Induce the stress-responsive gene	Improves dehydration and drought tolerance	[60]
10	*DREBs/* *CBFs*; *ABF3*	Stress-induced transcription factors	Enhanced expression of downstream stress-related genes confers drought tolerance.	[19]
11	*ZAT12*	Stress-induced transcription factors	Enhanced expression of downstream stress-related genes confers drought	[27]
12	*AtGAMT1*	Suppress gibberellin	*GAMT1* overexpression inhibited the expansion of leaf epidermal cells.	[61]
13	*SlNAC4*	Stress-responsive transcription factor	Modulation of ABA-independent signaling networks	[62]
14	*GalUR*	GalUR encodes Lgalactono- 1,4-lactone as a precursor of ascorbic acid	Ascorbic acid detoxifies superoxide anion radical and hydroxyl radical and also plays a crucial role in scavenging ROS	[63]
15	*EgDREB1*	Enhances the expression of DRE/CRT and DRE/CRT-containing genes	Elevated level of antioxidative enzymes scavenge the superoxide radical and accumulation of nonenzymatic antioxidants maintains the cell basic structure under drought and cold stress	[64]
16	*CcHRD*	AP2/ERF-like tanscritpion factor	Regulate many pathways involved in stress tolerance	[65]
17	*SlMAPK1*	Encodes for mitogen-activated protein kinases	*SlMAPK1* improves drought stress tolerance by activating antioxidant enzymes, reducing oxidative damage, and modulating transcription of stress-related genes.	[66]
18	*SiDHN*	Encodes for Dehydrins (DHNs) commonly hydrophilin LEA proteins	Increased chlorophyll a and b, carotenoid and relative water, proline and soluble sugar content and improve photosynthetic efficiency and suppress the formation of malondialdehyde H_2_O_2_ and O_2_	[67]
19	*MdSWEET17*	Sugar transporters	Enhances accumulation of sugars, such as glucose and fructose, which act as osmoprotestants and carbon source under drought stress	[68]
20	*SlSAMS1*	S-adenosylmethionine synthetase (SAMS)	SlSAMS1 modulates the production of polyamines and H_2_O_2_ and maintains the cellular homeostatasis	[69]
21	*AtDREB1A and BcZAT12*	Encodes for transcription factors	Independent expression of *AtDREB1A and BcZAT12* gene enhances drought tolerance in tomato	[14,23]
22	*SlGATA17*	Improves phenylpropanoid biosynthesis pathway activity	DNA-binding domain of GATA TFs regulates many pathways in plants and enhances drought stress tolerance	[70]
23	*CsECR*	Encodes for enoyl-CoA reductase (ECR) enzyme, which is involved in biosynthesis of cuticular waxes and catalyses the last step of very long-chain fatty acids (VLCFAs) elongation	Ectopic overexpression of CsECR increased the contents of total waxes and aliphatic wax leaves and fruits of the transgenic tomato and improves drought tolerance	[71]

## Data Availability

This study is a review article and contents no supporting data, all the literatures were cited within the manuscript from where the details were taken.
